# The Microbiological Memory, an Epigenetic Regulator Governing the Balance Between Good Health and Metabolic Disorders

**DOI:** 10.3389/fmicb.2018.01379

**Published:** 2018-06-26

**Authors:** Christian A. Devaux, Didier Raoult

**Affiliations:** ^1^IRD, APHM, MEPHI, IHU-Méditerranée Infection, Aix-Marseille University, Marseille, France; ^2^Centre National de la Recherche Scientifique, Marseille, France

**Keywords:** metabolic diseases, infectious diseases, microbiome, diet, dysbiosis, microbiological memory, epigenetic programming, gene expression

## Abstract

If the transmission of biological information from one generation to the next is based on DNA, most heritable phenotypic traits such as chronic metabolic diseases, are not linked to genetic variation in DNA sequences. Non-genetic heritability might have several causes including epigenetic, parental effect, adaptive social learning, and influence of the ecological environment. Distinguishing among these causes is crucial to resolve major phenotypic enigmas. Strong evidence indicates that changes in DNA expression through various epigenetic mechanisms can be linked to parent-offspring resemblance in terms of sensitivity to metabolic diseases. Among non-genetic heritable traits, early nutrition could account for a long term deviant programming of genes expression responsible for metabolic diseases in adulthood. Nutrition could shape an inadequate gut microbiota (dysbiosis), triggering epigenetic deregulation of transcription which can be observed in chronic metabolic diseases. We review herein the evidence that dysbiosis might be a major cause of heritable epigenetic patterns found to be associated with metabolic diseases. By taking into account the recent advances on the gut microbiome, we have aggregated together different observations supporting the hypothesis that the gut microbiota could promote the molecular crosstalk between bacteria and surrounding host cells which controls the pathological epigenetic signature. We introduce for the first time the concept of “microbiological memory” as the main regulator of the epigenetic signatures, thereby indicating that different causes of non-genetic heritability can interact in complex pathways to produce inheritance.

## Genetic and Non-Genetic Heritability

Although the genetic material of an individual can be considered as a highly stable element that may slowly evolve from one generation to the next by random mutations and selection pressure of the environment, the way this genetic material is used within the cell as part of the transcription process can vary in astonishingly different levels of plasticity. Beyond the outdated tendency assuming that only the DNA sequence is inherited across generations, data issued from genome-wide association (GWA) studies have shown that the most common human diseases transmitted by parents to their offspring, could not be explained by common genetic variants (Maher, 2008). This paved the way for the search for mechanisms that could account for the intergenerational transmission of phenotypic traits proved to be different from simple DNA sequences. The term “inclusive heritability” encompasses the genetic and non-genetic dimensions of inheritance. Several causes of non-genetic inheritable traits can be assigned to parental effect, adaptive social learning, influence of the ecological environment, and/or epigenetics (Danchin et al., 2011). Epigenetic modifications regulate through switch on or off the intensity and timing of gene expression without changes to the underlying DNA sequence. Epigenetic control plays a crucial role during embryologic development in the silencing of genes not required in specific cells (Wolffe and Matzke, 1999). Epigenetic mechanisms include DNA methylation (cytosine methylation at the CpG/cytosine-phosphate-guanine dinucleotide residues), histone modifications (histone packaging through acetylation, methylation, phosphorylation, biotinylation, ubiquitination, and ADP-ribosylation), chromatin remodeling (modification of chromatin architecture and restructuration of nucleosomes), and transcriptional or translation interference by non-coding RNA (ncRNAS including microRNAs, miRNAs involved in transcriptonal silencing; short -<30 nts- interfering RNAs, siRNAs involved in post-transcriptional silencing and chromatin condensation; piwi-interacting RNAs, piRNAS acting on transposon silencing; and long ->200 nts- non-coding RNA, lncRNAs influencing gene expression by forming complexes with chromatin-modifying proteins) (Egger et al., 2004; Peschansky and Wahlestedt, 2014). In recent years evidence was accumulated suggesting that epigenetic modifications can play a major role in the etiology of chronic diseases such as depression, obesity, and cardiovascular diseases (CVD) and this discovery was regarded as a scientific breakthrough (Shenderov and Midtvedt, 2014). More intriguing though was the observation that pathologic metabolic processes can be inheritable within a family, leading to the conclusion that epigenetic modifications could be inherited during cell division. A growing number of studies suggested that maternal and neonatal diet strongly influences epigenetic processes linked to chronic metabolic diseases (Choi and Friso, 2010). This prompted us to question whether the epigenetic changes can be somatically inherited and stays constant from one generation to another within the patient’s family or if it is the cell molecular microenvironment that remains stable between parents and children. According to the second hypothesis, the molecular microenvironment -let us provisionally say microbiota antigens- would lead to the conservation of such epigenetic signatures, making them appear heritable although potentially reversible. We hypothesize that early nutrition could shape an inadequate gut microbiota impairing microbiota antigens which, in turn, could account for a long term deviant programming of genes expression and progress toward chronic metabolic diseases during adulthood. We propose that the diversity of bacteria species defining the gut microbiota can be partly shared within patient’s family (because they shared the same diet for years) and that the molecular crosstalk between the microbiota, the trillions of bacteria that live within us, and host cells, shapes the epigenetic modifications found in the host and the offspring.

## Gut Commensal Microbiota and Risks Factors

Since a few decades, microbiologists have been convinced that our microbiota shapes gene expression. Yet, we are at the very first phase of discovery of mechanisms that lead bacterial species to alter expression of eukaryotic genes of the host and trigger metabolic diseases. Entering deeper into these multifactorial mechanisms could consist in investigating the molecular crosstalk between the microbiota and hosts cells.

**FIGURE 1:**
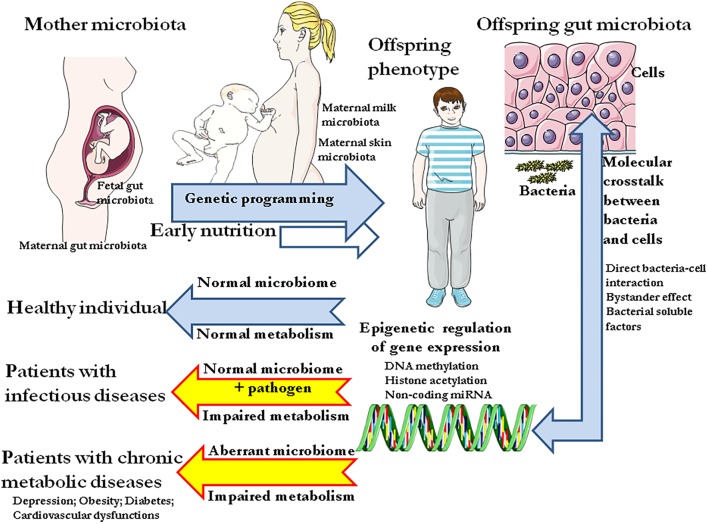
Schematic representation of the colonization of the gut by commensal bacteria since the fetal environment in the womb up to early period of life and from infants to adult individuals. The microbiota of infants and adults is essential to physiological metabolic processes (digestion) and should be capable of supplying the host with metabolic precursors, bioactive molecules neither primarily present in the diet nor produced by the host itself. The absorption of nutriments takes place at the level of the intestinal barrier, a vast epithelium surface of about 400 m^2^ maintained by tight junction between cells. Through constant molecular crosstalk with host cells, the gut microbiota can modulate the host metabolism by epigenetic regulation of cellular genes. Consequently, altered microbiota composition will provide aberrant signal to host cells resulting into metabolic disease.

Although the accumulation of data on the composition of the microbiota is becoming more precise every day, thanks to culturomics (Lagier et al., 2016), and genomics, there is still a long way to go to link microbiota and chronic metabolic diseases (sometimes called “civilization diseases”). However, it no longer remains questionable whether or not dysbiosis impacts cellular genes expression and progression toward metabolic diseases. In the 1990s, it became evident that prokaryotes played an important role in shaping gene expression in eukaryotic cells (Bry et al., 1996). Researchers have reported the importance of the fetal environment in the womb, and of postnatal colonization of the gut by commensal bacteria. Between the first and third trimester of pregnancy, there are shifts in maternal microbiota composition expected to confer evolutionary advantages for fecundity and infant survival (Koren et al., 2012). It is likely that the microbial colonization starts in the amniotic fluid and placenta and that the maternal gut supports the development of a prenatal microbiota (Collado et al., 2016). During vaginal delivery, the infant probably gain bacteria colonizing the maternal birth canal. To support this hypothesis, it could be argued that neonates born by C-section delivery exhibit aberrant gut colonization patterns (Dominguez-Bello et al., 2010). *Enterococcus faecalis*, *Staphylococcus epidermidis*, and *Escherichia coli* have been isolated from the meconium of healthy neonates, indicating that the fetus are far from being germ-free (Nicholson et al., 2012). Microbial contact during pregnancy and breastfeeding should also account for an important role in the selection of microbiota; during this early period of life, the infant’s gut will be colonized by health-promoting bacteria such as *Bifidobacterium* and *Lactobacillus* (Rautava et al., 2012). In the initial days of life, the gut microbiota is unstable and of low diversity (Arrieta et al., 2014). A microbiota with adult-like complexity is expected to be achieved at age of 3 years, when the infant’s diet evolves toward that of adult individuals (Yatsunenko et al., 2012). Methanogenic archaea (e.g., *Methanobrevibacter smithii* and *Methanosphaera stadtmanae*), that reduce hydrogen levels via production of methane thereby stimulating food fermentation, are not found during infancy while omnipresent in school-aged children (Van de Pol et al., 2017) (**Figure 1**). Interestingly, it was evidenced that Kwashiorkor patients (children with severe acute malnutrition) lack methanogenic bacteria (Million et al., 2016).

**FIGURE 2:**
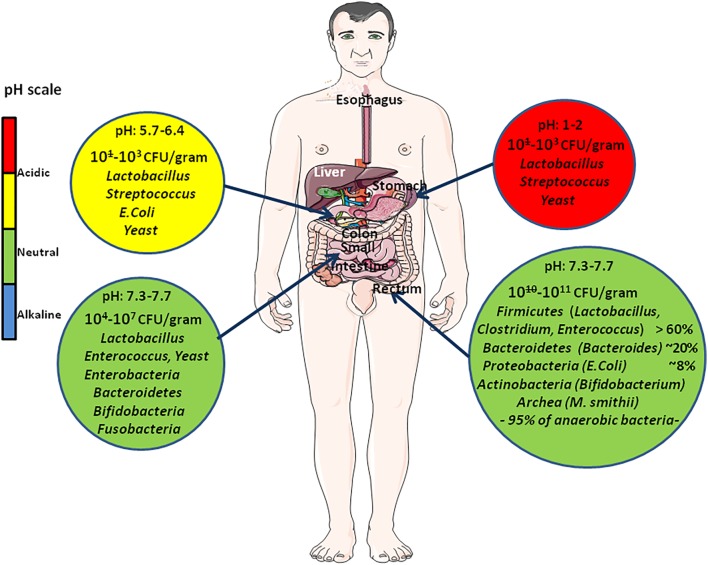
Quantitative and qualitative variation of intestinal flora all along the digestive tract. This diagram illustrates the variations in pH (that changes from acidic in the stomach to almost neutral or slightly alkaline in the intestine) and usual composition of the microbiome at different levels (stomach, small intestine, colon) of the digestive tract. The abundance of microbes is indicated as the number of colony forming unit (CFU) per gram. Due to the intimate crosstalk between gut microbiota and human cells, the gut microbiome integrity is of prime significance to human health.

**FIGURE 3:**
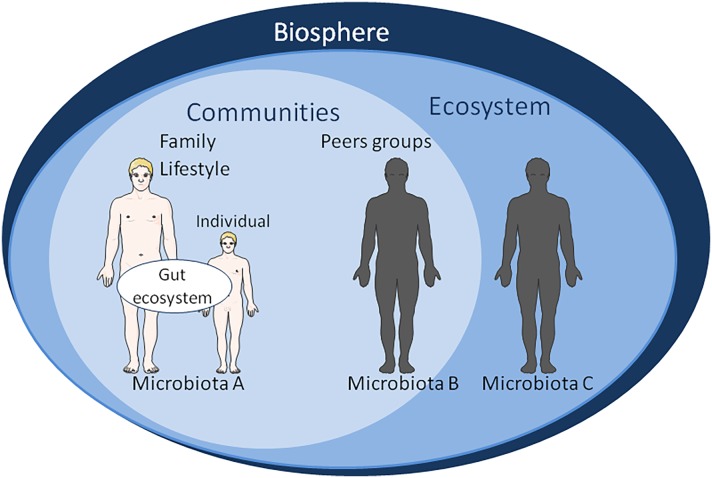
Schematic representation of factors influencing the gut microbiota ecosystem. At the level of biosphere: natural ecosystems, biodiversity, water resource, climate change. At the level of ecosystem: damage and loss of natural environment, biodiversity loss, land degradation, freshwater decline, air water pollution, population displacement. It is worth noting that the appearance of human on the planet has extensively changed the biosphere within a very short time on the evolution scale. At the level of community: human-made environment, natural ecosystems area, water resource, agricultural area, urban area, economic growth, sick-care system, responsible consumption, gender equality. Gut microbiomes from urbanized and pre-agricultural populations do not show the same bacterial strains’ diversity; At the level of family lifestyle: diet behavior, sexual behavior, psycho-socio-economic environment, physical environment, reduced environmental microbial biodiversity, reduced human contact with microbes, spiritual, culture. At the individual level: diet supplemented with probiotics or antibiotics, dysbiosis, systemic immune dysregulation, inflammatory disease, tissue damage, emotional, cognitive. The main vectors for transmission of biological information from one generation to the next are: (a) DNA sequence inheritance (mainly vertical inheritance from generation n to generation n+1). Genetic inheritance is vertical in humans. However, it is known that HGT is possible among microorganisms (e.g., bacteria and virus) and between microorganisms and plants; (b) Epigenetic inheritance (vertical inheritance). Phenotypic variation results from changes in DNA expression without changes of DNA sequence. We hypothesize that the molecular crosstalk between the “microbiological memory” and host cells shape epigenetic inheritance; (c) Parental effect (vertical inheritance). It refers to effects that parents have on the phenotypic traits of their offspring independently of the offspring’s own genotype; the offspring’s fitness is affected. It might be considered that nutrition shaping the microbiota of the offspring participates to non-genetic inheritance; (d) Adaptive social learning (involves both vertical and horizontal inheritance). It refers to information learnt from others and culturally transmitted that influences behavior. The human diet is usually mixed with meat, fruit, vegetables, water; the relative proportions of these compounds in diet have an impact on the microbiota. For various reasons, some families have a regular high-fat diet and/or very high sugar diet. Some groups of people choose to adopt a lacto-ovo-vegetarian diet, other are limited to lacto-vegetarian diet, and finally some even claim to be vegan. All these eating behavior alter the microbiota; and (e) Ecological environment inheritance (mainly vertical inheritance). Individuals act on their environment (named “environmental anthropization”) in such a way that it affects their fitness, thereby altering the selection pressures acting on them. We consider that “microbiological memory” belongs to this category of non-genetic inheritable phenotypic traits.

In adults, bacteria concentrations range from 10^1^–10^3^ per gram to 10^10^–10^11^ per gram in the upper intestines and colon respectively, and they reach their highest biomass in the distal gut (Hooper et al., 2001; Gill et al., 2006; Hugon et al., 2013). They are essential to physiological metabolic processes such as digestion and absorption of nutriments (**Figure 2**). A recent study has reported metagenomical and metatranscriptomical data providing evidence that the gut microbiome remains stable over time and that within a person, taxonomic and functional variation remain consistently lower than between persons (Mehta et al., 2018). A stable gut microbiota results from a long co-evolution process aimed at supplying the host with numerous metabolic precursors, bioactive molecules, cofactors, and signaling molecules, neither primarily present in the diet nor produced by the host itself. Within human bodies *Homo sapiens* DNA is estimated to account for less than 10% of the total DNA because of the incredibly large numbers of microorganisms that reside in (and on) humans, primarily within the gut (Human Microbiome Project Consortium, 2012). The characterization of microbiota has implied drawing maps of the most common elements of the gut microbiota. A considerable bacterial genetic diversity contributes to the microbiome’s steady state; with over 1,000 species and 7,000 strains at the human populations level, the human gut microbiota is an extremely complex ecosystem in which the phyla *Firmicutes* (species such as *Lactobacillus*, *Clostridium*, *Enterococcus*) and *Bacteroidetes* (species such as *Bacteroides*) account for the majority, though other phyla such as *Proteobacteria* (*Escherichia coli*), *Actinobacteria* (*Bifidobacteria*), *Cyanobacteria*, *Fusobacteria*, and *Verrucomicrobia* are also present in low abundance (Eckburg et al., 2005; Qin et al., 2010). Environmental selection and competitive exclusion between microbes during gut colonization, are expected to be the major driving forces that shape the core microbial diversity whereas stochastic factors and *in situ* evolution are likely at the origin of inter-subject variability among groups of people living in similar environments (Walter and Ley, 2011) (**Figure 3**). At the individual level, only a few hundred bacterial species are found. With its extremely high concentration of bacteria and bacteriophages, optimal temperature, the gut provides ideal conditions for horizontal gene transfer (HGT) (Lerner et al., 2017a). HGT enables the transfer of genetic materials by transformation (uptake of foreign genetic material), transduction (transfer of microbial DNA from one bacterium to another by a viral intermediate), and conjugal transfer (transfer of DNA via a mobile genetic elements). HGT, allows a rapid acquisition of a new function important to pass through the natural selection. The most documented HTG refers to dissemination of antibiotic resistance genes among bacteria. Yet, HTG from successfully adapted members of an ecosystem to other members of the ecological niche is common and likely increases the fitness of the recipients.

Advanced culturomics had made possible the search for new bacterial species in cohorts of individuals with a metabolic disease; namely *Urmitella timonensis*, *Bacillus andreraoultii*, *Blautia marasmi*, *Lachnoclostridium pacaense*, *Bacillus marasmi*, and *Anaerotruncus rubiinfantis* which were recently isolated from stool samples of undernourished African children (Pham et al., 2017). Several studies have established a relationship between the gut microbiota and the host’s metabolism (Bäckhed et al., 2004; Ley et al., 2005; Turnbaugh et al., 2006; Tidjani Alou et al., 2017). Other results point punctually to link a bacterial species with a metabolic disease; for example, after caloric restriction, high abundance of *Akkermansia muciniphila* and microbiome richness correlate with a healthier metabolic status in overweight/obese adult patients (Dao et al., 2016). In addition, a study that explored the fecal samples of 416 twin pairs in the United Kingdom found evidence that *Christensenella minuta* favored a low body mass index (Goodrich et al., 2014). The children born to malnourished Dutch women during World War II, were more likely to have chronic diseases like depression and diabetes, demonstrating the great correlation existing between the diet of a pregnant mother and expression of genes in her offspring (Choi and Friso, 2010). Since the pioneering works linking the microbiome to metabolic diseases, the search for the “microbial signature” of a metabolic disease has lead to an intense international scientific competition.

In parallel, major breakthroughs regarding the epigenetic signature of metabolic diseases have been made. Indeed, people who were exposed to famine *in utero* had a lower degree of methylation of a gene implicated in insulin metabolism (the insulin-like growth factor II gene) than their unexposed siblings (Heijmans et al., 2008). Currently, the number of scientific reports indicating that patients with metabolic diseases exhibit a particular epigenetic signature is steadily increasing. These data are opening new avenues for the investigation of relationships between microbiota and epigenetic programming in health and disease.

## Lessons From Clinics and Animal Models: Evidence of Links Between Gut Microbiota and Metabolic Diseases

### Emotional Disorders and Depression

Central nervous system (CNS) has long been considered an immune-privileged site capable of unidirectional signaling to the gut. It recently became obvious that the gut microbiome is also able to influence the brain via an array of multichannel sensing and trafficking pathways (Lerner et al., 2017b). Emerging evidence suggests that the gut microbiota may play a role in shaping cognitive networks that control the genesis of emotional and neurodevelopmental disorders such as depression, autism spectrum disorders, and schizophrenia (Kelly et al., 2017). Increased abundance of *Lactobacillus*, *Bifidobacterium* and *Ascomycota* in the oropharyngeal microbiota has been documented in patients with schizophrenia compared to healthy controls (Castro-Nallar et al., 2015). Maternal infection during pregnancy can be associated with the development of neurodevelopmental disorders (Jiang et al., 2015). Increased risk for mood, anxiety and depression disorders was reported after antibiotic exposure that alter the microbiome composition (Lurie et al., 2015). Clinically depressive episodes in humans are known to refer to dysregulation of the hypothalamic-pituitary-adrenal (HPA) axis (Barden, 2004). Using germ-free mice, a direct link between microbiota and the operation of the HPA axis was established based on the corticosterone and adrenocorticotrophin response to restraint stress (Sudo et al., 2004). In rats submitted to maternal separation stress, treatment with *Lactobacillus* spp. probiotics reduced stress responses (Gareau et al., 2007). Beside the modulation of the HPA axis, microbiota including bacteria such as *Citrobacter rodentium*, *Campylobacter jejuni, Bifidobacterium infantis*, or *E. coli*, may directly influence the CNS function and have a direct effect on c-FOS activation in cells from CNS (Foster and McVey Neufeld, 2013). A meta-analysis of infectious agents associated with schizophrenia has revealed that among others, *Chlamydophila pneumoniae* and *Chlamydophila psittaci* might be inducers of the disease (Arias et al., 2012). Another study indicated that *Lactobacillus*, *Tropheryma*, *Halothiobacillus*, *Saccharophagus*, *Ochrobactrum*, *Deferribacter*, and *Halorubrum* were increased whereas *Anabaena*, *Nitrosospira* and *Gallionella* were decreased during the first episode of schizophrenia (Schwarz et al., 2018). Species such as *Bacteroides vulgatus, Clostridium histolyticum* and *Clostridium bolteae* were reported to be over-represented in the gut microbiota of autists (Parracho et al., 2006; Finegold et al., 2010). SCFAs neurohormonal molecule (butyrate, acetate and propionate) are known to be produced by bacteria such as *Bacteroides*, *Bifidobacterium*, *Lactobacillus*, *Clostridium*, and *Prevotella* (Macfarlane and Macfarlane, 2012). Recently, a placebo-controlled trial showed that treatment with probiotic *Bifidobacterium longum* reduces depression scores but not anxiety, and increases the quality of life in patients with irritable bowel syndrome (Pinto-Sanchez et al., 2017). These observations indicate that the gut microbiota can act on the CNS.

### Inflammatory Bowel Disease (IBD), Crohn’s Disease (CD), Ulcerative Colitis, and Celiac Disease

The study of a cohort from Denmark has revealed that the incidence of IBD is significantly increased in patients born by C-section delivery and exhibiting aberrant gut colonization patterns, compared to controls born by vaginal delivery (Sevelsted et al., 2015). Enterotoxigenic *Bacteroides fragilis* has been suggested to be associated with acute and persistent diarrheal disease in patients with IBD and the oral intake of yogurt containing *Bifidobacterium longum* BB536 improves the clinical status of the patients (Odamaki et al., 2012). Using a model of mice infected with enterotoxigenic *B. fragilis*-inducing colitis, it was evidenced that induction of colonic hyperplasia and colorectal cancer is associated to the STAT-3 signaling pathway and a pro-inflammatory Th17 response (Wu et al., 2009). Very recently, by using the adenomatous polyposis coli tumor suppressor gene heterozygous APC^Min^ mice infected with enterotoxigenic *B. fragilis*, it was demonstrated that progression to cancer involves a multistep cascade requiring IL-17-dependent activation of NF-κB and STAT-3 signaling in colic epithelial cells that trigger chemokines expression which in turn activates mucosal Th17 response and distal colon tumorigenesis (Chung et al., 2018). A reduction in the diversity of microbiota species was recently reported in pediatric patients suffering from ulcerative colitis or CD. The microbiota of these young patients showed a low abundance of *Lactobacillus*, *Bifidobacteria*, *Eubacterium rectale*, and *Faecalibacterium prausnitzii* (Knoll et al., 2017). Fecal transplantation in the treatment of ulcerative colitis was recently considered as a promising rescue approach (Uygun et al., 2017). Interestingly, *Faecalibacterium prausnitzii* was shown to prevent the occurrence of experimentally induced inflammatory colitis in mice (Sokol et al., 2008). Adherent-invasive *Escherichia coli* are more frequently encountered in the intestinal mucosa of patients with CD than in healthy individuals (Darfeuille-Michaud et al., 2004). Moreover, it was reported that the stress response chaperon Gp96 is abnormally expressed at the apical surface of epithelial cells from the ileum of CD patients and acts as receptor for the outer membrane protein A (OmpA) of adherent-invasive *E. coli* thereby promoting colonization (Rolhion et al., 2010). Chronic inflammation linked to microbial infections can sometimes evolve toward cancer, as it has been demonstrated for gastric cancer associated to *Helicobacter pylori* infection (Sears and Pardoll, 2011), or *Coxiella burnetii*-induced lymphoma (Melenotte et al., 2016). Celiac disease, a polygenic autoimmune inflammatory disorder of the small intestine induced in genetically susceptible individuals following ingestion of gluten (a mixture of prolamin proteins present in wheat, rye, and barley) (Balakireva and Zamyatnin, 2016), could be included as a disease associated with the gut microbiota composition. Specific amino acid sequences in gluten activate T cells, which triggers inflammation. While gluten plays a major role in the induction of celiac disease, infection like *Campylobacter jejuni* in adults are associated with an increased risk of celiac disease, and a possible association between celiac disease and bacterial transglutaminase was also suggested (Lerner and Matthias, 2015). These data demonstrate that the gut microbiota can trigger inflammation and autoimmune diseases.

### Obesity (OBS)

A large Danish cohort investigation (Le Chatelier et al., 2013) revealed that non-obese and obese individuals differed in the richness of gut bacteria (58 species differing in abundance), and that people with low bacterial richness were characterized by weight gain over time, a marked overall adiposity, insulin resistance and dyslipidaemia. A study performed on a Danish cohort of 28,000 mother-child pairs, revealed that antibiotic exposure of children during the first 6 months of life was associated with increased risk of being overweight at age of 7 years (Ajslev et al., 2011). Another study investigating the effects of macrolide use in a cohort of Finnish children also concludes that early life antibiotic use resulted in disruption of the microbiome, long-lasting shift in microbiota composition with a depletion of *Actinobacteria*, increase in *Bacteroidetes* and *Proteobacteria*, and an increased risk for metabolic diseases (Korpela et al., 2016). Abnormal weight gain was a side effect observed in patients with long-term doxycycline and hydroxychloroquine treatment (Angelakis et al., 2014). It is worth noting that during the last 60 years, there has been a widespread use of antibiotics, such as tetracycline and penicillin, as food additives in mammalian livestock (pigs, cows, and sheep), poultry, and farmed fishes, with the multiple objectives of preventing and treating infectious diseases as well as promoting growth (Taylor and Gordon, 1955). Increase in *Proteobacteria* (*E. coli*) in pig gut microbiota was found after 2 weeks of a diet supplemented with antibiotic (Looft et al., 2012). The mechanism of weight gain in agricultural animals receiving food supplemented with antibiotics is not fully understood although it is likely the consequence of low bacterial richness in the gut microbiota. A sheep animal model showed that feeding mature females with food poor in folate, vitamin B12 and methionine during the periconceptional period induced obesity in adult offspring (Sinclair et al., 2007). In models of diet-induced OBS and genetically modified (ob/ob) mice, administration of a broad-spectrum antibiotic reduced weight gain (Cani et al., 2008) and improved glucose tolerance (Membrez et al., 2008). Finally, a very interesting observation is that a patient successfully treated with fecal microbiota transplantation for recurrent *Clostridium difficile* infection, developed new-onset OBS after receiving stools from a healthy but overweight donor (Alang and Kelly, 2015). It makes no doubt that there is a link between gut microbiota and weight.

### Diabetes Mellitus (DM)

Decline in abundance of the bacterial phyla *Firmicutes* and increase of *Bacteroidetes* in the gut microbiota, were correlated with the progression of type 1 DM, an autoimmune disorder that involves β-cell inflammation and destruction (Giongo et al., 2011). A case control study revealed that the gut microbiota of children with type 1 DM differs from that of healthy children with an increase in the abundance of *Bacteroidetes*, *Clostridium* spp. and *Veillonella* and reduction in *Lactobacillus*, *Bifidobacterium*, *Blautia*, and *Prevotella* (Murri et al., 2013). This observation corroborates an analysis comparing gut microbiota for type 1 DM, indicated an increase in the abundance of *Bacteroides* and *Akkermansia* and decrease in *Prevotella* in subject at high risk for diabetes (Alkanani et al., 2015). Decreased abundance of *Blautia* was also reported in a case-control study that examined feces from Chinese children with type 1 DM. In this study, a decreased abundance of *Lachnospira*, *Dialister*, and *Acidaminococcus* in the feces was also observed (Qi et al., 2016). It is known that maternal high fat feeding results in offspring with exacerbated adiposity and altered expression of proteins involved in hepatic insulin sensitivity (Buckley et al., 2005). In a rat animal model, feeding a protein-restricted diet to pregnant rats induced hypomethylation of the glucocorticoid receptor (Lillycrop et al., 2005), increased glucocorticoid receptor expression, and reduced the expression of the 11b-hydroxysteroid dehydrogenase type II enzyme which inactivates corticosteroids in the liver, lung and brain of the offspring (Bertram et al., 2001). In the liver, a higher expression of the glucocorticoid receptor increases phosphoenolpyruvate carboxykinase expression and activity and gluconeogenesis contributing to insulin resistance (Burns et al., 1997). Giving a protein-restricted diet to pregnant rats also increased glucokinase expression in the liver of the offspring and the capacity for glucose uptake (Bogdarina et al., 2004). Since five decades, converging evidence suggests that the frequency of type 1 DM is increasing in industrialized country to become a public health problem. Viral, environmental, chemical, life-style factors were suggested in the search for establishing causality. The hygiene hypothesis (Bach and Chatenoud, 2012) claims that the decline in infectious diseases resulting from better hygiene and medical care plays a major role in emerging metabolic/autoimmune diseases. For example, Tuberculosis is more frequent in the southern countries compared with those of the north and a negative correlation between the frequency of *Mycobacterium tuberculosis*-induced disease and type 1 DM has been clearly established (Airaghi and Tedeschi, 2006). According to the hygiene hypothesis, the difference in social and economic development between Finland and Russia which are neighboring countries, also account for the higher type 1 DM incidence in Finland compared with Russia (Kondrashova et al., 2007). The gut microbiota is modified in patients with type 1 DM.

### Cardiovascular Diseases

Almost four decades ago, in studying coronary artery disease death rates among 5,654 men born during 1911–1930 in Hertfordshire, United Kingdom, Barker and his colleagues observed that birth weight was inversely correlated with increased early death from ischemic heart disease (Barker et al., 1989). Furthermore, birth weight and rates of growth in the first 3 years of life have also been associated with adult onset of hypertension (Thompson, 2007). Yet, the Barker’s hypothesis is not supported by evidence from low-income countries, where intrauterine growth retardation and low birth weight are common but hypertension and coronary heart disease are less prevalent than in high-income countries; it could probably be explained by marked variations in microbiota composition between individuals from the north and those from the south. There has been evidence suggesting that alterations of gut microbiota may be associated to CVD (Tang et al., 2013). A study using high-throughput sequencing that compared the gut microbiota of 29 patients with coronary heart disease and that of 35 healthy volunteers indicated that the diversity and compositions of gut microbiota were different; the proportion of *Bacteroidetes* was lower and that of *Firmicutes* was higher in patients from the coronary heart disease group (Cui et al., 2017a). It was reported that the gut microbiota-dependent metabolite, trimethylamine *N*-oxide, whose levels are markers of CVD, could promote atherosclerosis (Tang and Hazen, 2014). A study consisting in the modulation of microbiota by supplementing probiotic *Lactobacillus plantarum* in the diet of a cohort of patients indicated that after treatment atherogenic parameters including LDL-cholesterol, were significantly reduced (Wu et al., 2017). These results were confirmed by the study of animal models. It was reported that lipoteichoic acid from *Lactobacillus plantarum* inhibits the inflammation causing atherosclerosis plaques by inhibiting NF-κB and activation of MAP kinases (Kim et al., 2013). A murine animal model showed that feeding mice with dietary supplementation of L-carnitine and choline, the precursor of trimethylamine *N*-oxide, accelerated atherosclerosis (Koeth et al., 2013). Interestingly, a study performed on mice genetically deficient in Toll-like receptor (TLR, a component of the anti-infectious innate immunity expressed in gut mucosa), indicated that these deficient mice developed metabolic syndrome, with body masses that were 20% greater than those of wild type mice, hyperlipidemia (increased serum level of triglycerides and cholesterol) and hypertension (Vijay-Kumar et al., 2010). Moreover, grafting “germ-free” mice with the microbiota from mice lacking TLR confers the sensitivity to metabolic syndrome to these mice. It was also found that deficiency of either TLR4 or Myd88 attenuates the high-fat diet-induced atherosclerosis and inflammation in apolipotrein E-deficient mice (Michelsen et al., 2004). It is also worth noting that a link between *Porphyromonas gingivalis* periodontal infections and CVD has been established (Pussinen et al., 2007). These reports evidence that a particular gut microbiota may be associated to CVDs.

## Food Shapes the Metabolome by Modifying the Microbiome

**FIGURE 4:**
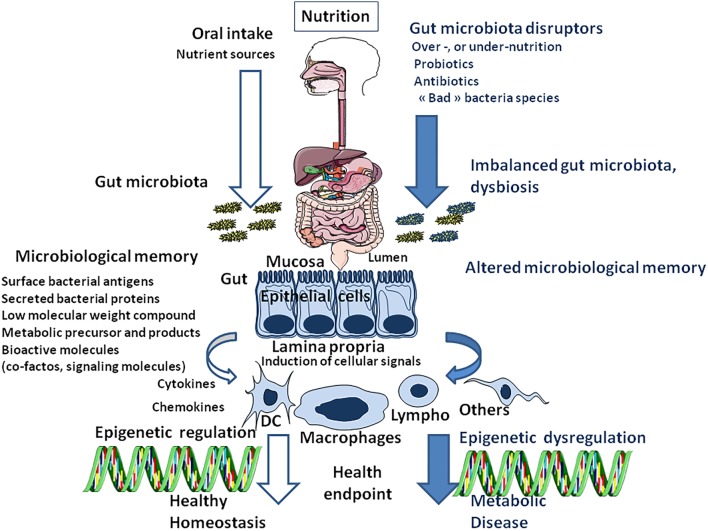
Schematic representation of nutrition/oral intake and the result on gut bacteria composition (microbiota) depending on the absence **(left)** or presence **(right)** of gut microbiota disruptors. Next, the cell microenvironment (including all surface antigens, secreted proteins and low-molecular-weight compounds from bacteria and bioactive molecules supplied through the diet and processed by the gut microbiota), named microbiota memory, trigger the activation of cellular signaling pathways that regulate gene transcription in different cells types [DC: Dendritic cells; Lympho: lymphocytes (B and T-cells);Macrophages; Others: other cell types]. Epigenetic programming is the result of a molecular crosstalk between gut prokaryotes (microbiota metabolome) and eukaryotic cells. When stable, this continuous reshaping of cellular genes appears as an epigenetic signature.

Metagenomic studies of the intestinal microbiome, have demonstrated that it is a dynamic entity, influenced by different factors, including diet and antibiotic treatment (**Figure 4**). During feeding, a very fast diet-microbiota crosstalk is established and it rapidly and reproducibly alters the human gut microbiota (David et al., 2014). Although bacteria essential to life are shared among people (Arumugam et al., 2011), there is a great diversity of bacterial species in the gut between related and non-related individuals. It seems that the presence or absence of some species in the commensal bacterial population predisposes to a risk of metabolic illness. This opens new avenues for research which is probably the very beginning of a scientific (and pharmaceutical) revolution. Studies using young (3 weeks old) “germ-free” mice, have shown that mice grafted with microbiota from mice treated with a low dose penicillin gained more weight and fat mass than mice grafted with microbiota from control animals. Also, mice grafted with microbiota from penicillin-treated mice showed reduced gut bacteria richness affecting species such as *Lactobacillus*, *Allobaculum*, *Rikenellaceae*, and *Candidatus arthromitus*, suggesting these bacteria might have a protective role in shaping adult metabolism (Cox et al., 2014).

Given that antibiotics are widely used for promoting growth in livestock, poultry and aquaculture, increasing levels of human food contamination by antibiotics have been reported. Meat and milk from these animals might contain traces of antibiotics which could modify the gut microbiota during early life (more vulnerable to change than later in life) and select antibiotic-resistant bacteria, with consequences on host cell metabolism and human health (i.e., increasing the risk of OBS in the general population) (Cox and Blazer, 2015). Studies in a “germ-free” chicken animal model, have shown that antibiotics alone demonstrated no growth-promoting effect (Coates et al., 1963), suggesting that antibiotics do not directly change the host’s metabolism but that the metabolic effects observed with antibiotic treatment in “normal” animals are driven by changes in their microbiota. In addition the effects of antibiotic treatment in the diet are not always predictable and their use is empirical. The administration of low doses of penicillin or oxytetracycline to mice was found to result in weight gain whereas high doses antibiotics resulted in weight loss (Dubos et al., 1963). It has also been claimed that *Lactobacillus* spp., *Streptococcus* spp., *Bacillus* spp., *Bifidobacterium* spp., *Enterococcus* spp. and other microbial species added in poultry feed, positively affect growth performance (Smith, 2014). Although many infant formulas already contain probiotics, it is likely that improving the knowledge on the function of “good” and “bad” bacteria will probably produce impact on the use of probiotics in human nutrition.

Moreover, food commensal bacteria are a potential important source for HGT (Wang et al., 2006), including probiotic bacteria which act as reservoir for antibiotic resistant determinants (Wong et al., 2015). It is estimated that about a quarter of all food production (e.g., ham, cheese, yogurt) involves bacterial fermentation processes using lactic acid bacteria and other microbial and fungal strains, opening the possibility of HGT among the bacteria of foodstuff (Aminov, 2010). In addition, microbial transglutaminases, which are functionally close to endogenous autoantigens involved in celiac disease, are extensively used as food additives. These molecules are used as cross-linking agents of proteins aimed at improving the texture of food products, and it has been suggested that microbial transglutaminases may be involved in the development of celiac disease (Lerner et al., 2017c). The increase of autoimmune diseases in humans is difficult to explain by genetic changes which may have occurred during the recent and relatively short period of human evolution. Thus, the role of introduction of industrially processed food in the onset of autoimmune diseases becomes increasingly recognized (Arleevskaya et al., 2017). It is currently hypothesized that industrially processed food may result in substantial loss of gut bacterial diversity contributing to these contemporary diseases (Mancabelli et al., 2017). Indeed hygienic behaviors which probably reduce microbial diversity associated with human gut colonization in early life, may compromise the establishment of appropriated immune responses thereby reducing the benefits of measure aimed at improving human health (Mulder et al., 2011). In particular, the long-term effect of antibiotic therapies in children under the age of 3 years should be evaluated.

## Epigenetic Programming/Reprogramming/Memory and Microbiota

Epigenetic programming is likely the integrated result of interactions derived from global metabolism, microbiota, immune system activation, and external factors such as diet (macro- and micro-nutriments), pharmaceuticals (particularly antibiotics), and environmental factors (pH, oxygen, temperature). Epigenetic developmental plasticity allows for a complex organism to adapt to microenvironmental signals, especially during early life, thereby increasing its fitness. The links between epigenetic programming and nutriments or microbiota have been investigated quite recently (Gallou-Kabani and Junien, 2005). Short chain fatty acids produced by bacteria have emerged as one clear link allowing microbiota to intersect with host epigenetic (Woo and Alenghat, 2017). Yet, most of the molecular mechanisms driving epigenetic modifications remain to be further explored. Food- or microbiota-derived folate (a water soluble B-vitamin), choline, betain and vitamin B12 might contribute to generating 6-methyltetrahydrofolate which is the methyl group donor for synthesis of SAM, a molecule involved in DNA methylation. Among others, *Lactobacillus* and *Bifidobacteria* produced folate, thereby affecting DNA methylation through regulation of methyl-donor (Rossi et al., 2011). In a sheep animal model, it was found that feeding mature females with food free of folate, vitamin B12 and methionine during the periconceptional period induced obesity in adult offspring and altered their methylation status (Sinclair et al., 2007). It has been suggested that folate may represent a good candidate for controlling DNA methylation since aberrant reprogramming of DNA methylation was observed after low dietary folate (Steegers-Theunissen et al., 2009). Histone methylation was also shown to be associated with OBS; the loss of Jhdm2a demethylase in mice results in OBS and hyperlipidemia (Tateishi et al., 2009).

An intriguing study reported that distinct DNA methylation profiles were found in blood samples from women 6 months after delivery, the result depended on the predominance of either *Firmicutes*, *Bacteroidetes*, and *Proteobacteria* in their fecal microbiota during pregnancy (Kumar et al., 2014). It was reported that inadequate intakes of long-chain polyunsaturated fatty acids (LCPUFAs) during pregnancy, may result in aberrant DNA methylation patterns in the offspring, thereby influencing their health (Khot et al., 2017). Increased maternal serum levels of vitamin B12 during pregnancy were found to correlate with a decrease in DNA methylation in newborns (McKay et al., 2012). Food might also provide molecules known to interfere with DNA methylation such as genistein. Butyrate that is produced by certain classes of bacteria, is a ligand for a subset of G protein-coupled receptors and also acts as a potent inhibitor of histone deacetylase (HDAC) (Bourassa et al., 2016). It was shown that mice with an intestinal epithelial cell specific deletion of the epigenome-modifying enzyme HDAC3 demonstrated alterations in the composition of the intestinal microbiota (Alenghat et al., 2013). It was also reported that most HDAC were downregulated in female mice with a high-fat diet (Panchenko et al., 2016). In addition, butyrate is capable of suppressing the activation (nuclear translocation) of NF-κB, a nuclear factor known to play a major role in immune response to infection (Iwai, 2012). It was reported that premalignant stages of gastric cancer associated to *Helicobacter pylori* were characterized by altered DNA methylation (Park et al., 2009) and histone acetylation patterns (Dinz et al., 2010). The relationship between *Helicobacter-*induced chronic inflammation, DNA damage and colon cancer was investigated using a model of Rag2^-/-^ mice infected by *Helicobacter hepaticus*. This showed that 5-Cl-dC mimics 5-methyl-dC induces inappropriate increases in CpG methylation that can silence tumor suppressor genes and initiate the carcinogenesis (Mangerich et al., 2012). Moreover, the most common gene mutated in colorectal cancer is the adenomatous polyposis coli (APC) tumor suppressor gene and the mechanisms of *APC* inactivation include hypermethylation of CpG sites in APC promotor and decreased translation due to inhibition by microRNA (Wu et al., 2012). Recently wild-type and TLR 2 knockout (Tlr2^-/-^) mice were used to examine the dynamic interplay between host and gut microbiota. DNA methylation patterns of the genes involved in immune response was found affected along with an altered composition of the mucosal microbiota (Kellermayer et al., 2011). It was reported that genetically modified gut TLR2 ligand lipoteichoic acid (LTA)-deficient *Lactobacillus acidophilus* suppress inflammation and protect mice against colitis (Mohamadzadeh et al., 2011) and colon cancer (Khazaie et al., 2012). By using a rat animal model it was observed that various phenotypic characteristics (OBS, cancer risk) could be obtained by changing the amount of vitamins, choline, betain and selenium in the diet of pregnant rat, and these changes were associated with increased CpG methylation of eukaryotic DNA (Delage and Dashwood, 2008). A mouse model, showed that gestational exposure to *Acinetobacter lwoffii*, triggers modulation of histone acetylation protecting the offspring from the development of asthma-like diseases (Brand et al., 2011). Overnutrition was reported to regulate the synthesis of several miRNAs whose expression control metabolism (Cui et al., 2017b). Finally, it was reported that specific diets (tea polyphenols, soybean genistein, or plant food isothiocyanates), might inhibit the development of cancer by reducing DNA hypermethylation in critical genes associated with cancer such as *p16 Ink* or *RAR*β (Fang et al., 2007). Since the diet strongly influences the composition of the microbiota, it is likely that the observed effects reflect, at least partly, the composition of the gut microbiota and that of their bioactive compound on cellular gene expression.

## Discussion

The gut microbiota is composed of diverse populations of commensal bacteria species that, together with salivary proteases, stomach secretions and gastric acidity, represent the first barriers of protection against colonization and invasion by pathogens (Pamer, 2016). During the past decades, the attention of infectious diseases physicians was focused on the identification of unique pathogens accidentally introduced into patients and causative agents of diseases. When the commensal bacteria failed to protect the host against pathogens and the immune system turned to be unsuccessful in eradicating the aliens on its own, the obligatory next step for the clinicians is identification of antibiotics to which the pathogen could be sensitive, for therapeutic use. Most frequently an antibiotic treatment intended to suppress adverse effects of the pathogens is sufficient to cure infected patients. When antibiotic treatments fail (e.g., antibiotic-resistant *Clostridium difficile* infection), fecal microbiota transplantation frequently shows its usefulness (Hocquart et al., 2017). This pragmatic vision of a fight against “bad” microorganisms has often been sufficient and successful in eradicating infectious pathogens such as OXA-48 carbapenemase-producing *Klebsiella pneumonia* or vancomycin-resistant *enterococci* (Lagier et al., 2015; Davido et al., 2017).

Although this “old-fashioned pathogen-suppression strategy” is clearly crucial in clinical treatment of patients suffering from infectious diseases, approaching bacteria-induced chronic metabolic disease with this perspective comes down to drastically simplify the problem. It deliberately ignored hundreds of commensal bacteria species whose presence in individuals is nothing less than essential to life as the result of the coevolution of hosts and their commensal microbiota. At the beginning of the XXIth century, the Human Microbiome Project from the National Institutes of Health (NIH HMP Working Group Peterson et al., 2009), was designed to repel the frontiers of microbiology by providing data, tools and resources aimed at understanding the role of changes in the resident microbiome in disease and health. During early postnatal life, humans are colonized by commensal intestinal bacteria, the gut microbiota is unstable during the first days of life and develops an adult-like complexity by age of 3 years. According to the Barker’s and the hygiene hypothesis, one might expect that common adult metabolic diseases are the results of how multiple gene were turned off or on to optimize perinatal and early adult life. Interestingly, the period of life during which epigenetic DNA imprinting is the most active overlap this early 3 years period (Bhutta et al., 2013). In healthy individuals, the gut microbiota forms stable communities of bacteria belonging to different species that display particular compositional and functional characteristics. Moreover, the microbiota remains relatively stable across generations of individuals within a family. In adults, bacterial concentrations range from 10^1^–10^3^ per gram in the upper intestines to 10^10^–10^11^ per gram in the colon. These bacterial concentrations should be compared to the 10^6^–10^7^ *Lactobacillus* spp. contained per gram of yogurt (Angelakis et al., 2011). This indicates that diets could theoretically modify the composition of the gut microbiota daily, thus changing the molecular microenvironment of host cells and consequently reshaping the eukaryotic genes by epigenetic reprogramming (Shenderov, 2012). Faced with these observations, it seem reasonable to consider that it is the relative stability of the diet that maintains the balance of the gut microbiota over time.

More recently, increasing evidence from metagenomic studies was reported indicating that the diversity of the intestinal microbiome differs from one individual to another, and that dysbiosis might determine risk factors. Microbial surface antigens and metabolic end-products can activate signaling pathways resulting in the modulation of the cells’ metabolome. It is worth noting that the lipopolysaccharide (LPS) receptor TLR4 which binds Gram-negative bacteria LPS, was found to promote diet-induced metabolic syndrome (Shi et al., 2006; Cani et al., 2008). This observation suggests that the molecular crosstalk between gut bacteria and host cells activates signaling pathways in host cells which will ultimately modulates gene expression through DNA-binding proteins activation and epigenetic modifications. LPS which is continuously produced within the gut by the death of Gram-negative bacteria and is carried into intestinal capillaries through a TLR4-dependent mechanism (Neal et al., 2006), is known to trigger the secretion of proinflammatory cytokines when it binds to the CD14/TLR4 receptor complex (Wright et al., 1990). In favor of the molecular crosstalk hypothesis, it was found that levels of *Bifidobacterium* spp. were reduced, whereas plasma LPS increased in mice given a high-fat diet for 4 weeks. Also, LPS infusion in wild type mice mimics the phenotype of mice given a high-fat diet (Backhed et al., 2007). Moreover, mice given a high-fat diet for 8 weeks developed a LPS receptor-dependent vascular inflammation (higher thoracic aorta IκBα-phosphorylation, ICAM, IL-6). Finally, it was reported that the mechanism underlying the resistance to diet-induced obesity in germ-free mice, depends on the activation of AMP-activated protein kinase (Cani et al., 2007). Obviously, bacteria have acquired multiple systems to expose proteins at their surface and to release soluble factors in the extracellular environment, over the course of evolution. More global approaches are therefore required to study the metabolome from gut bacteria and deciphering their functional participation in cellular gene modulation linked to chronic diseases (Maffei et al., 2017). Within families of patients with chronic metabolic diseases, bacterial induction of phenotypic changes in the offspring that persist for life implies stable modifications to gene transcription and altered activities of metabolic pathways. It has been known for a long time that patients with bowel disease had increased risks of colorectal cancer, starting with the formation of flat lesions or polyps protruding from the bowel wall that progress toward dysplastic adenomas and colic carcinoma (Couturier-Maillard et al., 2013). Recent studies indicate that it is feasible to rescue an experimental model of colic cancer by oral treatment using genetically engineered bacteria. This is only one among numerous demonstrations that reconstitution of a favorable microenvironment may be advantageous for treatment of metabolic diseases. Interventional changes in the composition of the microbiota (oral administration of commensal bacteria) can lead to reshape the prokaryotic molecular effectors of the eukaryotic microenvironment. This binary classification opposing bacteria that favor the development of diseases to others that protect against their occurrence is likely oversimplified since it ignores the dual behavior of certain pathobionts. It was recently reported that the translocation to the liver and other systemic tissues of *Enterococcus gallinarum* that usually live as a symbiont in the gut, can promote autoimmunity in mice and humans (Vieira et al., 2018), opening a new avenue for research. Moreover, it was recently shown that efficacy of cancer immunotherapy with immune checkpoint antibodies requires specific gut microbiota and is decreased by antibiotic treatments (Zitvogel et al., 2018).

**FIGURE 5:**
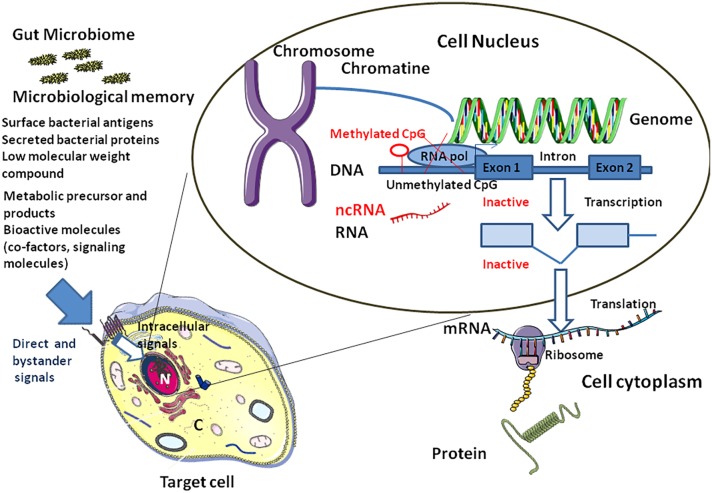
Link between microbiota and epigenetic modifications. The “microbiological memory” will deliver direct and bystander signals to the target cell (left panel). After cell surface interaction (e.g., soluble bacterial compound interacting with a cell surface receptor complex), intracellular signal activation pathways will be modulated (e.g., phosphorylation of cytoplasmic proteins by kinases, nuclear translocation of transcription factors,…), thereby influencing the balance between activation and genetic silencing of transcription by DNA methylation and ncRNAs. Nucleosomes (not shown) comprises an octamer of histones and double-stranded DNA. When CpG dinucleotides are unmethylated in the gene promoter region, the RNA polymerase (RNA pol) can bind and activate transcription **(right)**. Methylation of CpG dinucleotides (red symbols) by DNA methyl transferases will recruits a histone deacethylase (HDAC)/histone methyl transferase (HMT) complex which in turn will remove acethyl groups from histones and methylate specific residues the overall effect being silencing of transcription. Transcriptional and/or translational interference by non-coding RNA (ncRNAs) is also illustrated.

We are just beginning to understand that the intestinal microbiota is a dynamic system that readjusts daily according to nutritional intake. Yet, little is known about the distant effects of these bacteria on eukaryotic epigenetic regulation. Recently, differential DNA methylation and covalent modification of histones that regulate gene transcription were associated to nutrition. Both under- and over-nutrition during pregnancy and/or lactation were shown to induce stable modifications of the offspring and termed “fetal programming” with a connotation of genetic inheritance. The link between microbiota and epigenetic modifications should be further investigated. Here, we hypothesize that when epigenetics shows inherited characteristics (so-called “epigenetic programming”), it is actually the cell microenvironment (bacterial surface antigens and secreted proteins, low-molecular-weight compound from bacteria and bioactive molecules supplied through the diet and processed by the gut microbiota), that remains constant from one generation to the next. We propose the term “microbiological memory” to account for this microbiota-shaped microenvironment (**Figure 5**). Microbiological memory would remain stable when diet and microbiota remain almost unchanged. According to this model, what is currently known as epigenetic programming is probably nothing more than a non-genetic inheritable signature resulting from the molecular crosstalk between gut prokaryotes (microbiota metabolome) and eukaryotic cells. This crosstalk would trigger a continuous reshaping of cellular genes through activation of signaling pathways in host cells, thereby controlling the epigenetic signature. It should be emphasized that this remains a hypothesis and, as such, has to be verified in future studies. While epigenetic can be investigated in a more or less straightforward manner by studying diseases’ signature, the influence of the microbiological memory is much more difficult to decipher because of the involvement of many variables. Studies are very much needed to discriminate what is causal and what is co-occurrence in the trinomial diet-microbiota-epigenetics.

## Author Contributions

CD and DR contributed to the conception of the manuscript. CD wrote the manuscript.

## Conflict of Interest Statement

The authors declare that the research was conducted in the absence of any commercial or financial relationships that could be construed as a potential conflict of interest.
